# Recognizing Decline and Supporting Care Transitions in Older Adults: Homecare Nurse Perspectives

**DOI:** 10.1093/geroni/igaa057.1682

**Published:** 2020-12-16

**Authors:** Suzanne Sullivan, Catherine Mann, Samantha Mullen, Yu-Ping Chang

**Affiliations:** University at Buffalo, Buffalo, New York, United States

## Abstract

Older adults with serious illness residing in the community are at risk for decline and death. Homecare Registered Nurses (RNs) are in an ideal position to recognize serious illness and engage older adults and their caregivers in discussions about goals for care, while guiding transitions to supportive care services such as palliative or hospice care. However, little is known about this process, or how homecare RNs act upon this information. Using a grounded theory approach, data were collected through focus group interviews with 35 RNs working in homecare. A social process rooted in relationship-based care over time was identified using the constant comparative method. RNs recognize serious illness and support care transitions by identifying changes in illness trajectories and assessing the impact of such changes on quality-of-life, adapting and accommodating care to support older adults in the home for as long as possible, communicating with the care team, engaging stakeholders, and maneuvering through complex systems of care; ultimately relinquishing care to other providers and settings. Our findings also reveal that RNs feel inadequately prepared and frustrated with a fragmented healthcare system and lack of collaboration among the team in supporting the best care transition for older adults and their caregivers. Our findings reinforce the importance of promoting care continuity in homecare settings whenever possible, suggesting a critical need to develop training and team processes that support and empower RNs, so that they may lead care transitions as changing needs emerge during serious illness management of older adults.

